# Deep brain stimulation for Parkinson's disease in Brazil: equity, timing, and systems-level responsibility

**DOI:** 10.1055/s-0046-1824559

**Published:** 2026-07-14

**Authors:** Renato Puppi Munhoz

**Affiliations:** 1 UHN, Toronto Western Hospital, Edmond J. Safra Program in Parkinson's Disease and Morton and Gloria Shulman Movement Disorders Clinic, Toronto, Ontario, Canada.; 2 University of Toronto, Department of Medicine, Division of Neurology, Toronto, Ontario, Canada.; 3 Krembil Brain Institute, Toronto, Ontario, Canada.

Deep brain stimulation (DBS) is recognized as an established therapy for Parkinson's disease (PD) with robust evidence for benefit, medication burden reduction, and safety in high-volume centers worldwide. 1 Yet, the diffusion of this technology remains a fractured landscape, mirroring global inequities in health systems' capacity, financing, and workforce distribution. 2 3 Hence, the real-world experience from a Brazilian center caring for PD patients through the public health system, published in this issue of the Arquivos de Neuropsiquiatria, exposes, through seemingly conflicting lenses, the views of DBS both as a promise and a hurdle, with structural barriers that prevent timely and universal access to it in proper time. 4

The authors analyzed a cohort of 101 PD patients who underwent DBS between 2012 and 2024, illustrating how a high-complexity public center can deliver a sophisticated therapy even under resource constraints. 4 Although more direct outcomes measures of motor benefit are not presented, a surrogate marker, namely, the reduction in levodopa equivalent daily dose, achieved 25% at 12 months, indicating a meaningful decrease in medication burden for patients and caregivers. 4 Serious complications were infrequent, comparable to those reported in previous international series. When read in concert, this experience contradicts any lingering perception that cutting-edge interventions cannot be implemented with quality in Latin American public hospitals; the limitation lies not in technical capacity but in scale and organization. 3 4 Against this backdrop, the timelines reported are a stark reminder that access delayed is often benefit denied. On average, patients underwent DBS almost 11 years after diagnosis and more than two years after surgical indication, despite evidence that earlier intervention can optimize outcomes and possibly slow functional decline. 2 5 This represents a tapestry woven from many threads: a single center serving a large region, restricted operating-room time for functional neurosurgery, bottlenecks in preoperative evaluation, and funding ceilings that limit the number of procedures. 2 6 Also, disease progression during this waiting period may erode the therapeutic window that justified DBS in the first place.

Another finding that demands a systems-level reading is the high frequency of battery depletion events and the delays in generator replacement. 4 In this cohort, 9.9% of patients experienced complete battery depletion, and some required hospitalization because of abrupt stimulation withdrawal, including a case of DBS withdrawal syndrome. 4 7 These events are not mere technical hiccups, they are sentinel warnings of a fragile supply chain and financial support model for advanced therapies in general. When battery replacement depends on limited quotas or even legal action initiated by patients, the health system offloads risk onto those most vulnerable. 6 8 Preventing avoidable stimulation interruption should be viewed as part of ethical obligations that accompany the decision to implant a device at the outset. 8

The demographic profile of the operated patients also raises important questions about equity by presenting a cohort of predominantly white males, in a country where unfair racial and social disparities in health access are well documented. 6 8 While the study was not designed to disentangle causal pathways behind these distortions, the pattern aligns with broader literature showing that complex, elective procedures tend to be disproportionately accessed by individuals with higher socioeconomic capital, better education, and stronger health literacy. 2 4 8 If DBS is to be considered part of the therapeutic arsenal of a universal health system, then monitoring and correcting access disparities must become a deliberate policy objective rather than an incidental observation. This Brazilian series is particularly valuable as it moves beyond individual efficacy to highlight health-system performance around DBS in a middle-income context. 4 Low surgical volume (<1 procedure/month over 12 years), long delays between indication and surgery, and dependence on non-systematic pathways for hardware replacement are not isolated institutional issues; they reflect how functional neurosurgery is suboptimally positioned within public-sector logistical planning and budgeting. 6 8 Unfortunately, despite the growing burden of PD and evidence for the cost-effectiveness of the procedure when compared with pharmacotherapy alone, DBS remains either technically unavailable or functionally inaccessible in many countries. 3 6 8

What should change? First, DBS programs in public systems need to be explicitly integrated into healthcare structures that match regional demands with capacity for procedural care. 2 3 8 This includes defining minimum surgical volumes, protecting dedicated operating-room time for functional neurosurgery, and structuring multidisciplinary teams. Second, financing models must evolve from individual procedure-based approvals to integrated coverage across the lifecycle of the implanted system, including programming, streamlined battery replacement, etc., aiming to create efficient maintenance models assuring that each new implantation will be properly monitored to prevent the risk of catastrophic therapy interruption a few years down the line. 7 9 Third, addressing inequities in access requires proactive engagement and adequate referral pathways. 6 8 Neurologists in non-tertiary-care settings need greater awareness not only about the criteria and benefits of DBS but also about the importance of timely referral, before irreversible disability or comorbidities preclude surgery. 1 10 Waiting lists should be monitored with the inclusion of equity variables (race, gender, socioeconomic status, geographic origin, etc.), so that systemic biases can be detected and mitigated. 2 6 8 Finally, the inclusion of Latin American cohorts in international DBS consortia would help ensure that guidelines and prognostic models also include and reflect the realities of diverse health systems.

The present report, anchored in a public hospital in southern Brazil, shows that DBS for PD can be delivered safely and effectively within the public health system, but also exposes where the system is failing: timeliness, continuity, and fairness of access. 4 The ethical question for policymakers is no longer whether DBS “works” but whether societies are willing to organize their health systems so that the benefits of this technology are distributed in a timely and equitable manner. 2 Latin America, which combines universal coverage aspirations and relentless resource constraints, is uniquely positioned to serve as an example worldwide of how a high-complexity therapy like DBS can be scaled in a way that honors both clinical efficacy and social justice. 2 3 Doing so will require more than isolated centers of excellence and goodwill; it will demand systematic and aligned policies with the common goal to generate population level benefit unaffected by constraining variables under universal, publicly financed care.
